# Adverse Events and Hospital-Acquired Conditions Associated With Potential Low-Value Care in Medicare Beneficiaries

**DOI:** 10.1001/jamahealthforum.2021.1719

**Published:** 2021-07-23

**Authors:** Kelsey Chalmers, Valérie Gopinath, Shannon Brownlee, Vikas Saini, Adam G. Elshaug

**Affiliations:** 1 Lown Institute, Needham, Massachusetts; 2 Menzies Centre for Health Policy, Sydney School of Public Health, University of Sydney, Australia, NSW; 3 Centre for Health Policy, Melbourne School of Population and Global Health, The University of Melbourne, Australia, VIC

## Abstract

**Importance:**

There has been insufficient research on the patient harms and costs associated with potential low-value procedures in the US Medicare population.

**Objective:**

To report the prevalence of adverse events associated with potential low-value procedures and the additional hospital length of stay (LOS) and costs.

**Design, Setting, and Participants:**

This is a retrospective cohort study using Medicare fee-for-service claims between January 2016 to December 2018. Participants were aged 65 years or older. Procedures were selected if they had previously published indicators of low-value care, including knee arthroscopy, spinal fusion, vertebroplasty, percutaneous coronary intervention (PCI), carotid endarterectomy, renal stenting, and hysterectomy for benign conditions. Analysis was conducted from July to December, 2020.

**Main Outcomes and Measures:**

For inpatient procedures, the number and rate of admissions with a hospital-acquired condition (HAC) or patient safety indicator event (PSIs), as well as the unadjusted and adjusted difference in mean LOS and Medicare costs between admissions with and without a HAC/PSI. For outpatient procedures, we report the number of claims where the beneficiary had an unplanned hospital admission within seven days and the number of these admissions with a HAC/PSI.

**Results:**

There were 573 351 patients included in the study, with 617 264 procedures; the mean (SD) age was 74.2 (6.7) years, with 320 637 women (55.9%), and mostly White patients (520 735; 90.8%). Among the 197 755 claims for the inpatient procedures, 231 had an HAC and 1764 had a PSI. Spinal fusion was associated with the most HACs (123 admissions) and PSIs (1015 admissions). Overall, HACs during a PCI admission were associated with the highest adjusted additional mean LOS (17.5 days; 95% CI, 10.3-23.6), with also the highest adjusted additional mean cost ($22 000; 95% CI, $9100-$32 600). There were 419 509 included outpatient procedures, and 7514 (1.8%) had an unplanned admission within 7 days. A total of 17 HACs and PSIs occurred in these admissions.

**Conclusions and Relevance:**

In this cross-sectional cohort study of Medicare fee-for-service claims, patients receiving potential low-value care were exposed to risk of unnecessary harm associated with higher cost and LOS.

## Key Points

**Question:**

What is the prevalence and cost of hospital-acquired conditions (HACs) and patient safety events (PSIs) associated with procedures that may be low value?

**Findings:**

In this retrospective claims analysis of a cohort of Medicare fee-for-service beneficiaries, there were 231 HACs and 1764 PSIs in 197 755 claims for 7 inpatient procedures from 2016 to 2018.

**Meaning:**

Patients with flagged, potential low-value procedures were harmed while in hospital, resulting in an extended length of stay and additional costs.

## Teaser

This retrospective cohort study examines the prevalence of adverse events associated with potential low-value procedures and the additional hospital length of stay and costs.

## Introduction

The World Health Organization estimates that 1 in 10 patients in high-income countries are harmed while receiving hospital care.^1^ Ideally, the benefits of treatment outweigh this risk, but this may not be the case for patients receiving low-value care. Numerous studies have estimated the prevalence of low-value care using claims data. Whereas the volume and financial costs are one concerning aspect of low-value care, another aspect is the resulting physical harms, which may be unnecessary if the care was avoidable.^2^ Our objective was to estimate the inpatient adverse events (and their additional costs) to Medicare beneficiaries who likely received a low-value procedure.

Overuse or low-value care is the delivery of tests and procedures that provide little clinical benefit, increase health care spending without improving health outcomes, or risk patient harm in excess of potential benefits.^3^ This care contributes $75.7 billion to $101.2 billion per year in health care costs, based on estimates reported in gray literature and peer-reviewed publications between 2012 to 2019.^4^ An estimated 42% of Medicare beneficiaries received 1 or more low-value services per year.^5^

Despite these high costs and volumes, progress on reducing low-value care has been slow.^6^ One issue is that patients and clinicians might overestimate the treatment benefit relative to the probability of harm.^7^ Further, by only reporting on volumes and costs of low-value care, reduction efforts are often framed as a cost-cutting or rationing exercise.^2,8^ Few studies have reported the harms of low-value care using administrative data. An Australian study^9^ reported the incidence of hospital acquired conditions (HACs) solely occurring during public hospital admissions with a low-value procedure, and found between 0.2% and 15% of these patients had a HAC reported during their admission.

Our aim in this study was to report adverse events associated with procedures that met established low-value indicators in the US Medicare population. These estimates indicate which procedures (of those with low-value administrative data algorithms) have the highest hospital-acquired harm counts, and could direct investigation efforts on potentially reducing procedure rates based on patient safety, rather than total costs. We report adverse events using HACs and the Agency for Health Care Research and Quality (AHRQ) patient safety indicators (PSIs), which we selected given the Centers for Medicare & Medicaid Services (CMS) uses these to measure and compare hospital rates. The Centers for Medicare & Medicaid Services implemented the HACs Initiative in 2008 and denies incremental payment for admissions with specific HACs.^10^ Patient safety indicators are defined using a numerator (the adverse event) and a denominator (at-risk patients), and hospitals with higher PSI rates relative to other hospitals may face financial penalties under the CMS HAC Reduction Program.^11^

We report the PSIs and HACs for admissions with procedures meeting established low-value care criteria, and estimate the effect of these events as the additional costs and length-of-stay (LOS) for these admissions. We also estimate how many outpatient procedures had an associated, unplanned inpatient admission within 7 days and whether a HAC/PSI occurred during these admissions.

## Methods

### Data

We used a 100% sample of CMS 2016 to 2018 outpatient and MedPAR data. Reported race and ethnicity was defined by the Race of Beneficiary variable in the Master Beneficiary Summary File.

We included claims for Medicare fee-for-service (parts A and B) beneficiaries who resided in the US and were aged 65 years or older on the claim date. Inpatient claims were only included if they were from hospitals paid under the inpatient prospective payment system (IPPS), which are required to report present on admission (POA) diagnosis flags (necessary for the PSI/HAC algorithms). Outpatient claims were excluded if beneficiaries had a change in coverage or died within 7 days of the claim date.

This study was approved and received a waiver of patient consent by the New England institutional review board because this was secondary data analysis and low risk to participants.

### Low-Value Measures

We used previously published measures of low-value care if they were for procedures likely to be the main reason for a hospital visit.^5,12,13^ These procedures were knee arthroscopy, spinal fusion, vertebroplasty, percutaneous coronary intervention (PCI), carotid endarterectomy (CEA), renal stenting, and hysterectomy for benign conditions.

Inpatient procedures were identified through *International Statistical Classification of Diseases and Related Health Problems, Tenth Revision (ICD-10)-Procedure Coding System* and *ICD-10-Clinical Modification* codes in MedPAR. Outpatient procedures were identified either in the outpatient revenue center claims (via *CPT* codes) or in the outpatient claims table (via *ICD* codes). Two procedures, CEA and PCI, required previous condition histories (stroke and ischemic heart disease) from the Master Beneficiary Chronic Condition Segment file. The procedure algorithms are documented in the eTable in the Supplement.

We excluded inpatient claims if the service code was not the recorded principal procedure (that is, the main reason for the admission). We excluded claims for PCI or CEA if the beneficiary did not have Parts A and B coverage in the previous year because the low-value indicators of these services required a previous history of claims. The eFigure in the Supplement describes the final study cohort.

### Outcomes

#### Inpatient Admissions: Hospital-Acquired Conditions and Patient Safety Indicators

We selected 11 HACs and 6 PSIs listed in the Box.^14,15,16^ This included HACs relevant to included procedures (for example, we did not report HAC-11: surgical site infection for bariatric surgery). We included PSIs if they were relevant to the procedures, did not measure death rates, and had a high numerator count (at least 2000 cases) in the AHRQ 2019 publicly reported cases in the Medicare population.^17^ Each HAC/PSI algorithm was defined using criteria recorded in MedPAR, with a HAC/PSI event occurring if a complication is recorded without a POA flag. For each HAC/PSI reported result, we only included claims that fit the inclusion/exclusion criteria for that algorithm. We compared PSI rates with the 2019 AHRQ reported Medicare population rate as a cursory check of whether these rates were substantially higher or lower than this population.^17^

Box. **Included Hospital-Acquired Conditions and Patient Safety Indicators**

**Hospital-Acquired Conditions**

- Foreign object retained after surgery
- Air embolism
- Blood incompatibility
- Stage III and IV pressure ulcers
- Falls and trauma
- Catheter-associated urinary tract infection
- Vascular catheter-associated infection
- Manifestations of poor glycemic control
- Surgical site infection—certain orthopedic procedures of spine, shoulder, and elbow
- Surgical site infection following cardiac implantable electronic device procedures
- Iatrogenic pneumothorax with venous catheterization

**Patient Safety Indicators**

- Stage III or IV (or unstageable) pressure ulcers (PSI-3)
- Iatrogenic pneumothorax (PSI-6)
- Perioperative hemorrhage or hematoma (PSI-9)
- Postoperative respiratory failure (PSI-11)
- Perioperative pulmonary embolism or deep vein thrombosis (PSI-12)
- Postoperative sepsis (PSI-13)

#### Costs and Bed-Days

We reported the difference in mean LOS and cost between admissions with and without an associated HAC/PSI. We used linear regression to adjust LOS and cost for age, sex, similar diagnosis-related group (DRG) and comorbidity (using the ARHQ Elixhauser comorbidity score) differences between admissions with and without a HAC/PSI.^18,19^ Similar DRGs were mapped from the admission DRG by collapsing related DRGs with and without complication or comorbidities. We used bootstrapped samples (N = 1000) to report confidence intervals for our results that accounted for the potential hierarchy in the data at the hospital level.^20^ We used the total Medicare payment amount and pass through amount for an admission as the Medicare cost.^21^

#### Outpatient Procedures: Unplanned Hospital Admission Within 7 Days

As a large proportion of the investigated procedures occurred in the outpatient setting, we investigated whether any of these procedures were associated with an unplanned hospital admission within 7 days and the HACs/PSIs during these admissions. Our definition for this outcome was the numerator of CMS’s unplanned 7-day inpatient admission after outpatient surgery (excluding emergency department or observation stays that did not result in a MedPAR claim). This algorithm labels admissions within 7 days of the service date as ‘planned’ if the principal procedure code relates to an organ transplant, chemotherapy or rehabilitation, or a potentially planned procedure (that is, a procedure that is usually nonemergency) without an acute condition or complication principal diagnosis code.^22^ All other admissions within 7 days were considered unplanned. We excluded those that were not at IPPS hospitals before measuring HACs/PSIs.

Claims analysis was performed using SAS Enterprise Guide statistical software (version 7.15 HF8, SAS Institute Inc.) on the CMS Virtual Research Data Center and R (version 4.0.3, R Foundation). Analysis was performed from July to December 2020. We followed the Strengthening the Reporting of Observational Studies in Epidemiology (STROBE) reporting guideline for cross-sectional studies.^23^ The study protocol was registered on the Open Science Framework, https://osf.io/2egak/.

## Results

There were 573 351 patients included, with 617 264 total procedures in our sample with 197 755 (32.0%) as inpatient and 419 509 (68.0%) in outpatient. The mean [SD] age of included patients was 74.2 [6.7] years, with 320 637 women (55.9%), and mostly White patients (520,735; 90.8%).

### Inpatient Indicated Low-Value Procedures

Of the 197 755 admissions with a low-value procedure, 231 (0.1%) had at least 1 HAC and 1764 (0.9%) had at least 1 PSI. Table 1 and Table 2 show the HAC and PSI rates per procedure.

**Table 1.** Inpatient Procedures (That Were Also the Principal Procedure of the Admission) and the Recorded Number of Hospital-Acquired Conditions (HACs), the Difference in Mean Length of Stay (LOS), and Medicare Cost

Service	Inpatient procedures, No.	HACs, No.	HACs per 1000 procedures	HACs per 1000 bed days	Difference in mean LOS with and without HAC (days)		Difference in mean cost with and without HAC ($1000)	
					Unadjusted	Adjusted^a^	Unadjusted	Adjusted^a^
Spinal fusion	97 246	123	1.3 (1.0-1.5)	0.3 (0.3-0.4)	9.4 (7.7-11.3)	9.4 (7.5-11.1)	21 (15.9-26.8)	19.7 (14.9-24.4)
Vertebroplasty	20 833	60	2.9 (2.2-3.6)	0.5 (0.4-0.7)	8 (4.5-12.3)	6.9 (3.6-10.7)	8.3 (3.9-15.0)	4.2 (1.4-6.7)
PCI	12 864	19	1.5 (0.9-2.3)	0.5 (0.3-0.7)	19.6 (12.9-26.8)	17.5 (10.3-23.6)	46.7 (14.8-94.7)	22.0 (9.1-32.6)
Hysterectomy	17 283	14	0.8 (0.4-1.3)	0.3 (0.2-0.5)	7 (2.7-12.2)	3.7 (−0.7-9.0)	23.6 (6.0-45.2)	4.2 (−3.1-13.6)
CEA^b^	45 501	<11	NC	NC	NC	NC	NC	NC
Knee arthroscopy^b^	2010	<11	NC	NC	NC	NC	NC	NC
Renal stenting^b^	2018	<11	NC	NC	NC	NC	NC	NC

Abbreviations: CEA, carotid endarterectomy; NC, not calculated; PCI, percutaneous coronary interventions.

^a^ Adjusted for age, sex, similar diagnosis related group code and Elixhauser comorbidity scores using linear regression.

^b^ Rates, LOS, and costs are not reported for services where fewer than 11 admissions had an HAC.

**Table 2.** Inpatient Procedures (That Were Also the Principal Procedure of the Admission) and the Recorded Number of Patient Safety Indicators (PSIs), the Difference in Mean Length of Stay (LOS), and Medicare Cost

Service	Inpatient procedures, No.	PSIs, No.	PSIs per 1000 procedures	PSIs per 1000 bed days	Difference in mean LOS with and without PSI (days)		Difference in mean cost with and without PSI ($1000)	
					Unadjusted	Adjusted^a^	Unadjusted	Adjusted^a^
Spinal fusion	97 246	1015	10.4 (9.6-11.2)	2.8 (2.6-3)	6.4 (5.8-7.0)	6.4 (5.8-6.9)	22.7 (20.8-24.8)	21.3 (19.5-23.1)
CEA	45 501	470	10.3 (9.3-11.4)	6.9 (6.2-7.6)	3.5 (3.0-4.1)	3.1 (2.6-3.6)	10.2 (8.5-12.3)	8.1 (6.9-9.1)
Hysterectomy	17 283	142	8.2 (6.8-9.8)	3.4 (2.8-4.0)	5.5 (4.1-6.9)	4.8 (3.5-6.1)	10.7 (7.3-15.1)	8.0 (5.5-10.7)
PCI	12 864	103	8.0 (6.4-9.6)	2.6 (2.1-3.0)	7.0 (4.9-9.2)	6.3 (4.5-8.3)	18.5 (10.3-29)	12.0 (7.1-16.7)
Vertebroplasty	20 833	67	3.2 (2.5-4)	0.6 (0.4-0.7)	4.9 (3.0-7.2)	4.4 (2.6-5.5)	9.7 (5.4-15.3)	6.7 (4.3-8.2)
Renal stenting	2018	27	13.4 (8.1-19.3)	2.1 (1.3-3.0)	9.4 (4.3-15.2)	6.9 (3.0-10.5)	28.2 (9.2-50.4)	10.7 (2.8-19.4)
Knee arthroscopy^b^	2010	<11	NC	NC	NC	NC	NC	NC

Abbreviations: CEA, carotid endarterectomy; NC, not calculated; PCI, percutaneous coronary interventions.

^a^ Adjusted for age, sex, similar diagnosis related group code and Elixhauser comorbidity scores using linear regression.

^b^ Rates, LOS, and costs are not reported for services where fewer than 11 admissions had an HAC.

The procedure associated with the most HACs was spinal fusion (120 admissions with a HAC, 51.9% of all HACs), followed by vertebroplasty (60 admissions, 26.0%). The most frequent HACs were catheter-associated urinary tract infections (79 admissions, 33.8% of all HACs) and falls and trauma (74 admissions, 31.6% of all HACs). Spinal fusion had the highest number of PSIs (974 admissions with a PSI, 55.2% of all admissions with a PSI), with the second highest was CEA (452 admissions, 25.6%). The most frequent PSIs were PSI-9: perioperative hemorrhage or hematoma (552 admissions, 30.1% of all PSIs) and PSI-12: perioperative pulmonary embolism or deep vein thrombosis (467 admissions, 25.5%).

Table 3 reports the individual PSI rates across admissions with the included procedures. Perioperative hemorrhage/hematoma (PSI-9) for CEA had the highest rate (319 PSIs of the 44 835 included CEA procedures; 7.1 per 1000 CEA procedures; 95% CI, 6.5-7.7). Generally, PSI rates were lower than the 2019 AHRQ reported PSI rates, with the exception of the PSI-9 rates for CEA, renal stenting, and PCI and PSI-6 (iatrogenic pneumothorax) rates for spinal fusion procedures.

**Table 3.** Patient Safety Indicator (PSI) Rates Per 1000 Admissions, Where the Admission Fit the PSI Denominator Criteria

Procedure	PSI-03: Pressure ulcers	PSI-06: Iatrogenic pneumothorax	PSI-09: Perioperative hemorrhage or hematoma	PSI-11: Postoperative respiratory failure	PSI-12: Perioperative pulmonary embolism or deep vein thrombosis	PSI-13: Postoperative sepsis
AHRQ rate^a^	0.55	0.26	2.38	7.44	3.89	5.18
Spinal fusion	0.25 (0.15-0.34)	0.27 (0.18-0.36)	1.47 (1.27-1.67)	3.01 (2.65-3.33)	3.53 (3.17-3.86)	2.64 (2.34-2.96)
CEA^b^	NC	NC	7.11 (6.48-7.74)	2.58 (2.17-3.02)	NC	0.69 (0.48-0.87)
Hysterectomy^b^	NC	NC	1.82 (1.33-2.28)	3.27 (2.54-4.01)	2.38 (1.79-2.92)	1.23 (0.82-1.62)
PCI^b^	NC	NC	4.01 (3.09-4.91)	NC	1.25 (0.75-1.75)	3.60 (2.61-4.60)
Vertebroplasty^b^	NC	NC	NC	NC	2.44 (1.89-2.95)	NC
Renal stenting^b^	NC	NC	6.37 (3.42-8.91)	NC	NC	NC

Abbreviations: AHRQ, Agency for Health Care Research and Quality; CEA, carotid endarterectomy; NC, not calculated; PCI, percutaneous coronary interventions.

^a^ AHRQ-reported rate for 2019 Medicare population older than 65 years.

Estimates are shown for services if the observed PSI count was 11 or higher over 2016 to 2018.

^b^ Rates, LOS, and costs are not reported for services where fewer than 11 admissions had an HAC.

The total LOS for admissions with a low-value procedure was 978 980 days. For admissions with HAC, there was an estimated 2272 additional days between the total observed LOS (3161 days) and the adjusted predicted LOS without a HAC. Admissions with a PSI were associated with 10 331 total additional days (3444 days per year). Table 1 and Table 2 show the unadjusted and adjusted difference in mean LOS for admissions with and without a HAC/PSI. Adjustment reduced these differences between the 2 admission types. Hospital-acquired conditions were associated with an increase in mean LOS for all admissions, ranging from 3.7 days (adjusted) for hysterectomy to 17.5 days for admissions with a coronary stent (Table 1). Patient safety events were also associated with an increase in mean LOS for all admissions (Table 2), ranging from 3.1 days (adjusted) for CEA to 6.3 days for PCI.

Overall, HACs were associated with a total additional cost of $3.18 million (or $1.06 million per year), and PSIs were associated with $26.68 million ($8.89 million per year). Table 1 and Table 2 show differences in mean cost for admissions with a HAC/PSI and those without. Admissions with a HAC ranged from an additional mean adjusted cost of $4200 for vertebroplasty (an increase of 17.2% from the mean admission cost without an HAC) to $22 000 for PCI (42.8% increase), whereas admissions with a PSI ranged from an additional mean adjusted cost of $6700 for vertebroplasty (27.2% increase) to $21 300 for spinal fusion (60.3% increase).

### Unplanned Admissions After an Indicated Low-Value Outpatient Procedure

Of 419 509 outpatient low-value procedure claims, 7514 were associated with at least 1 likely unplanned admission within 7 days at IPPS hospitals (1.8% of the total outpatient visits); Table 4 shows these counts by procedure. We observed just 17 HAC and PSI events in 7744 unique admissions. Outpatient spinal fusion was associated with the highest rate of unplanned admissions (4.8 admissions per 100 outpatient services). Patient safety events and/or HACs occurred during the unplanned admissions for patients with a previous outpatient hysterectomy, knee arthroscopy, PCI, spinal fusion, and vertebroplasty.

**Table 4.** Outpatient Procedures With a Likely Unplanned Inpatient Admission Within 7 Days

Service	Outpatient procedures, No.	Unplanned admissions within 7 days		
		Admissions, No.	Admissions per 100 procedures	Inpatient bed-days per 100 procedures
Spinal fusion	61 365	2946	4.8 (4.4-5.2)	27.6 (24.5-31.2)
PCI	142 730	2161	1.5 (1.4-1.6)	5.7 (5.3-6.1)
Vertebroplasty	53 456	1533	2.9 (2.7-3.1)	19.9 (17.8-22.1)
Hysterectomy	54 036	472	0.9 (0.8-1)	3.8 (3.3-4.3)
Knee arthroscopy	99 127	390	0.4 (0.4-0.4)	1.7 (1.5-2)
Renal stenting	8464	213	2.5 (2.1-2.9)	7.7 (6.1-9.2)
CEA^a^	331	NC	NC	NC

Abbreviations: CEA, carotid endarterectomy; NC, not calculated; PCI, percutaneous coronary intervention.

^a^ Rates, LOS, and costs are not reported for services where fewer than 11 admissions had an HAC.

## Discussion

To our knowledge, this is the first study to estimate hospital-acquired harms for US Medicare beneficiaries associated with low-value care. We found a total of 239 HACs and 1773 PSIs; about less than 1% of admissions with an indicated low-value procedure. For inpatient admissions, HACs cost an estimated additional $1.06 million and 757 additional days in hospital per year, whereas PSIs were associated with $8.9 million and 3444 additional hospital days per year. Overall, these numbers are quite small relative to the size of the Medicare budget and number of beneficiaries, and also to the number of procedures that met the low-value measure criteria. These safety events, particularly HACs, are rare in the general Medicare population, so the low counts are not altogether surprising.

There are several high-priority candidates for further investigation and targeted efforts for reduction, based on their volume and the effect of the adverse events. Admissions with spinal fusion had the highest counts of HACs and admissions with vertebroplasty had the greatest rate. Percutaneous coronary intervention, although associated with a lower volume of HACs compared with vertebroplasty, had a higher adjusted increase in mean LOS and cost (a similar amount to spinal fusion). Spinal fusion was associated with the highest volume of PSIs, the second highest increase in LOS, and highest cost for admissions with and without a PSI (adjusted).

Most PSI rates for these procedures were lower than the overall Medicare population PSI rates.^17^ This may be because patients with these procedures may be healthier than the general Medicare inpatient population, particularly if high-risk patients are discouraged from receiving low-value procedures. In their study of general surgical and vascular surgical patients, Cima et al^24^ observed that PSIs have varied sensitivity among different groups of patients compared with the ACS National Surgical Quality Improvement Program (NSQIP) measures.

An Australian study reported between 0.2% to 15% of patients with a low-value procedure had an HAC during the admission.^9^ Although HAC measures in Australia and the US both serve a similar purpose (neither Australian state governments nor CMS pay for the higher DRG cost of these never events), the estimated range of HACs in Australia is higher than what was observed in our study. Australia reports different and likely more frequent HACs compared with the US, including unplanned intensive care unit admissions and medication complications.^9^ The Australian study also included all hospital patients older than 18 years and investigated procedures with measures not applicable to a Medicare cohort.

### Limitations

One limitation of our study is the specificity of low-value care indicators.^25^ Schwartz et al^5^ used 2 indicator versions to demonstrate the possible range of results from different low-value care definitions and algorithms. Two of the procedures in our study (hysterectomy for benign conditions and spinal fusion) were from the John Hopkins Overuse Index,^12^ which has been used to indicate overuse at regional levels. These algorithms may underestimate or overestimate true counts of low-value care, and have not been compared with a standard such as medical chart review. We did convert these algorithms from *ICD-9* to *ICD-10* procedure and diagnosis codes, which likely improved the specificity of the algorithms due to added precision in *ICD-10* codes.

The procedures included in this study represent a fraction of services that may be routinely overused and may lead to downstream harm. There has been a groundswell of studies documenting new low-value care measures, but most are for unnecessary tests and imaging.^26,27,28^ Mafi et al,^29^ using the measures from the Milliman MedInsight Health Waste Calculator, reported that the high volume of low-value testing made up the bulk of wasted financial costs they observed. Some studies have investigated cascade events (additional health care use or new diagnoses) after a low-value test, but the question of how to attribute all resulting patient harm and additional health care use to an index low-value procedure remains open (that is, beyond the hospital admission).^30,31^

The HAC and PSI outcome measures are also a limitation in this study. Although CMS uses these measures to penalize underperforming hospitals, there are validity issues with these claim-based algorithms. A systematic review by Winters et al^32^ found few studies comparing HACs or PSIs to medical chart records, and only 5 measures had enough published data to be included in the authors’ meta-analysis. Only 1 measure met the authors’ validity threshold of a positive predictive value of 80% (this was PSI-15: accidental puncture and laceration, not included in our study because it did not apply to our investigated procedures). Results from other data sets (and populations) vary: Horn et al^33^ reported at least 3% of patients with elective spine surgery in the American College of Surgeons’ NSQIP data set had at least 1 HAC, which is higher than our observed rate.

We reported the potential unplanned admissions within 7 days of potential low-value outpatient procedures. While these admissions are indicative of complications from an outpatient procedure, our applied measure did not allow for specific exclusions of acute conditions that may be unrelated to the procedure, so we expect the true rate of related inpatient admissions to be lower. For example, if a person had an outpatient knee arthroscopy and was soon after admitted for an unrelated condition, this would be counted as an unplanned admission associated with the outpatient procedure. Within the admissions we did report, however, counts of HACs/PSIs were small. The true counts of HACs/PSIs in admissions that are a direct result of a complication from an outpatient procedure are likely very low.

The HAC and PSI measures do not include some very serious events known to be associated with our investigated procedures. Measuring these events, however, would likely require creation of new algorithms or further development of existing algorithms for both low-value procedures and selected adverse events; that could potentially require more information than what is available in claims data. For example, CEA poses a risk of stroke during the procedure, a risk that is only outweighed by the potential benefits for those patients who already have a high risk of stroke within 5 years.^34^ The low-value CEA algorithm excludes both patients with a history of stroke and those with a stroke code in the claim. That means that patients who received a potential low-value CEA who suffered an intraoperative or perioperative stroke would not be counted by the algorithm either as an instance of a low-value service or as a harm associated with a low-value service. Defining new measures that are more sensitive to capturing these adverse events will be necessary for future research on harms associated with low-value care.

## Conclusions

In this cross-sectional cohort study of Medicare fee-for-service claims, we found HACs/PSIs in about 1% of inpatient admissions with a potential low-value procedure. Although discussions and efforts to address low-value care have started to focus more on patient harms and safety,^35^ there is clearly a need to develop more specific measures of low-value procedures and related harms to assess their effect. In addition, beyond the direct physical harm we investigated, there are many dimensions to potential harms from low-value care other than physical (such as psychological and financial harms) as outlined by Korenstein et al.^36^ This study has focused on a small fragment of the potential physical consequences of low-value care. Development and use of patient safety measures in claims data could further demonstrate these harms, which may help frame the issue of avoiding low-value care as a patient safety issue and encourage efforts to promote high-value care.

## Notes

Supplement. eFigure. Study Cohort Selection eTable. Low-Value Procedure Description and Codes eReferences
