# Supplementary material for: Adverse Events and Hospital-Acquired Conditions Associated With Potential Low-Value Care in Medicare Beneficiaries
Source: JAMA Health Forum. 2021 Jul 23;2(7):e211719. doi: 10.1001/jamahealthforum.2021.1719 (PMC8796970; doi:10.1001/jamahealthforum.2021.1719)
Supplement: Supplement. — eFigure. Study Cohort Selection eTable. Low-Value Procedure Description and Codes eReferences [file jamahealthforum-e211719-s001.pdf]

Supplemental Online Content

Chalmers K, Gopinath V, Brownlee S, Saini V, Elshaug AG. Adverse events and hospital-acquired conditions associated with potential low-value care in Medicare beneficiaries. *JAMA Health Forum*. 2021;2(7):e211719. doi:10.1001/jamahealthforum.2021.1719

eFigure. Study Cohort Selection
eTable. Low-Value Procedure Description and Codes
eReferences

This supplemental material has been provided by the authors to give readers additional information about their work.

© 2021 Chalmers K, et al. *JAMA Health Forum.*

eFigure. Study Cohort Selection

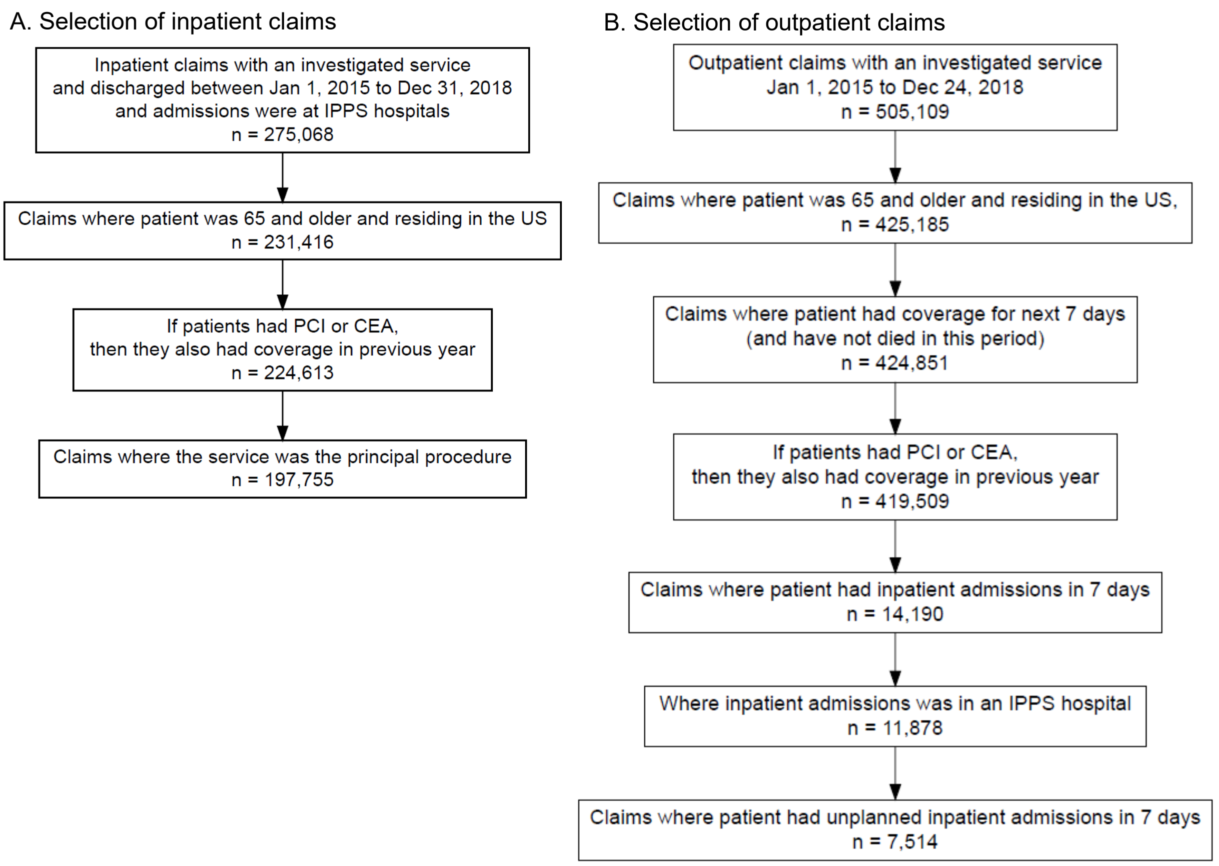

eFigure 1. Cohort inclusion for A) patients with an inpatient low-value procedure, and B) patients with an outpatient low-value procedure. IPPS: Inpatient prospective payment system; PCI: percutaneous coronary intervention; CEA: carotid endarterectomy.

© 2021 Chalmers K, et al. *JAMA Health Forum.*

eTable. Low-Value Procedure Description and Codes

We based these definitions and algorithms those reported in Schwartz et al[1,2] and Segal et al[3], further described in Chalmers et al[4]. Here, we have updated these algorithms to ICD-10 from ICD-9 using the CMS general equivalence mapping tables and manual review of the codes.[5] We also adapted these to fit a hospital-level indicator: CPT codes previously found in carrier claims were instead found in the outpatient revenue tables, or with the equivalent ICD-10 procedure code in outpatient or MedPar.

All codes are available on our Open Science Framework page: https://osf.io/2egak/?view_only=5451b92ebfd442e1b64baea885b7966f.

eTable 1. Low-value procedure description and codes

Service definition	Algorithm description
Carotid endarterectomy (CEA) for patients without a history of stroke or transient ischemic attack (TIA) and without stroke, TIA, or focal neurological symptoms noted in claim. Operation not associated with an emergency room (ER) visit.	CEA ICD-10-PCS codes in outpatient or MedPar, or CEA CPT code in outpatient. CEA ICD-10-PCS codes map to *Extirpation of Matter from* [Left/Right] [Position] *Carotid Artery* [Approach]. CPT: 35301.<br><br>Exclude service if ICD-10-CM codes associated with stroke, TIA or focal neurological symptoms (R20x, R25x, R26x, R27x, R29x, R414, R43x, R47x, R683, Z8673, G45x, I60.9, I61.9, I63.019, I63.119, I63.[019, 119, 139, 20, 219, 22, 239, 330, 240, 350, 359], I66.[09, 19, 29], I66.9, I67.848, I67.89, I97.811) in claim.<br><br>Exclude service if a patient's first date of indication of Stroke/Transient Ischemic Attack from Chronic Conditions Warehouse (CCW) table is not empty and prior to the service.<br><br>Exclude service if a patient had any ER visits within 14 days prior: flagged by CPT codes (99281 - 99285); MedPar admission type flag is 1 (emergency); the source of inpatient admission code is 7 (ER); ER charge amount is greater than zero; or outpatient revenue centre codes (045[0 - 2, 6, 9], 0981).
Hysterectomy for benign disease	Hysterectomy ICD-10-PCS codes in outpatient or MedPar, or CPT codes in outpatient. ICD-10-PCS codes map to *Resection of Uterus* [Approach].<br><br>Exclude service if malignancy codes are in claim: C51x - C58x, C7982; or carcinoma in situ codes: D06x - D07x.

© 2021 Chalmers K, et al. *JAMA Health Forum.*

Service definition	Algorithm description
	Exclude service if Diagnosis Related Group (DRG) in MedPar related to malignancy: 734 - 756.
Inferior vena cava (IVC) filter	IVC filter ICD-10-PCS codes in outpatient or MedPar, or CPT code in outpatient. ICD-10-PCS codes map to *Insertion of Intraluminal Device into Inferior Vena Cava,* [Approach].
Arthroscopic debridement/ chondroplasty of the knee with diagnosis of osteoarthritis or chondromalacia in the procedure claim	Arthroscopy ICD-10-PCS codes in outpatient or MedPar, or CPT code in outpatient. ICD-10-PCS codes map to *Inspection of* [Left/Right] *Knee Joint, Percutaneous Endoscopic Approach*; *Excision of* [Left/Right] *Knee Joint, Percutaneous Endoscopic Approach; Repair of* [Left/Right] *Knee Bursa and Ligament, Percutaneous Endoscopic Approach; Repair of* [Left/Right] *Knee Joint, Percutaneous Endoscopic Approach; Release of* [Left/Right] *Knee Joint, Percutaneous Endoscopic Approach; Repair of* [Left/Right] *Patella, Percutaneous Endoscopic Approach.*<br><br>Include service if osteoarthritis (M17x, M1991) or chondromalacia (M224x, M9420, M9426x, M9429) is in claim.
Coronary stent placement or balloon angioplasty for patients without unstable angina. Procedure not associated with an ER visit, which might be indicative of acute coronary syndrome.	ICD-10-PCS codes in outpatient or MedPar, or CPT code in outpatient. ICD-10-PCS codes map to *Dilation of Coronary Artery,* [One - Four Arteries], [Device Type], *Percutaneous Endoscopic Approach.*<br><br>Include service if the first indication date of Ischemic Heart Disease from the CCW data is at least 6 months prior to the service.<br><br>Exclude service if any unstable angina (I200, I25110, I25700, I25710) or myocardial infarction (I21x, I22x, I23x) codes in claim or within outpatient or Medpar within the previous 14 days.<br><br>Exclude service if a patient had any ER visits within 14 days prior: flagged by CPT codes (99281 - 99285); MedPar admission type flag is 1 (emergency); the source of inpatient admission code is 7 (ER); ER charge amount is greater than zero; or outpatient revenue centre codes (045[0 - 2, 6, 9], 0981).
Renal/visceral angioplasty or stent placement for patients with hypertension, diagnosis of renal atherosclerosis or	ICD-10-PCS codes in outpatient or MedPar, or CPT code in outpatient. ICD-10-PCS codes map to *Dilation of* [Left/Right] *Renal Artery with* [Device Type], [Approach].

© 2021 Chalmers K, et al. *JAMA Health Forum.*

Service definition	Algorithm description
renovascular hypertension, and no diagnosis of fibromuscular dysplasia of renal artery, in procedure claim.	Include service if renovascular hypertension is in claim (I150, I701, I773).<br><br>Exclude service if fibromuscular dysplasia (I773) is in claim.
Spinal fusion on lower vertebrae, excluding patients with radicular symptoms, herniated disc, radicular pain and scoliosis.	ICD-10-PCS codes in outpatient or MedPar, or CPT code in outpatient. ICD-10-PCS codes map to *Fusion of* [Lumbar, Lumbosacral, Thoracolumbar, or Thoracic] *Vertebral Joints* (including two or more), [Device], [Approach]; *Replacement of* [Lumbar or Lumbosacral] [Joint/Disc] with [Substitute type], [Approach].<br><br>Exclude service if there were two occurrences of radiculopathy (M541x) or sciatica (M543x) codes within 30 days (in MedPar, outpatient or carrier claims) prior to the procedure, or postlaminectomy syndrome (M961).
Vertebroplasty for vertebral fracture for patients with osteoporosis.	ICD-10-PCS codes in outpatient or MedPar, or CPT code in outpatient. ICD-10-PCS codes map to *Supplement* [Cervical, Thoracic, Lumbar or Sacrum] *with Synthetic Substitute,* [Percutaneous Endoscopic or Percutaneous] *Approach.*<br><br>Include service if osteoporosis pathological fracture code (M8000x, M8008x, M8080x, M8088x) in claim. Otherwise, include service if vertebra fracture code (S220x, S320x, S321x) and patient has Osteoporosis indicator in CCW table prior to the service.<br><br>For principal procedure exclusions in MedPar/inpatient, services were not excluded if the principal procedure was a kyphoplasty. These ICD-10-PCS codes mapped to *Reposition of* [Cervical, Thoracic, Lumbar or Sacrum], *Percutaneous Approach*.

© 2021 Chalmers K, et al. *JAMA Health Forum.*

eReferences

1. Schwartz AL, Landon BE, Elshaug AG, Chernew ME, McWilliams JM. Measuring Low-Value Care in Medicare. *JAMA Intern Med*. 2014;174(7):1067-1076. doi:10.1001/jamainternmed.2014.1541
2. Schwartz A. Replication Code/Data for Medicare Low-Value Service Measurement. Published online 2017. Accessed January 15, 2021. https://doi.org/10.7910/DVN/DEW0UO
3. Segal JB, Nassery N, Chang H-Y, Chang E, Chan K, Bridges JFP. An Index for Measuring Overuse of Health Care Resources With Medicare Claims. *Medical Care*. 2015;53(3):230-236. doi:10.1097/MLR.0000000000000304
4. Chalmers K, Smith P, Garber J, et al. Assessment of overuse of medical tests and treatments at US hospitals using Medicare claims. *JAMA Network Open*. 2021;Submitted.
5. Centers for Medicare & Medicaid Services. 2018 ICD-10 CM and GEMs. Published 2017. Accessed January 15, 2021. https://www.cms.gov/Medicare/Coding/ICD10/2018-ICD-10-CM-and-GEMs

© 2021 Chalmers K, et al. *JAMA Health Forum.*
